# Laparoscopic Versus Open Partial Nephrectomy: A Systemic Review and Meta-Analysis of Surgical, Oncological, and Functional Outcomes

**DOI:** 10.3389/fonc.2020.583979

**Published:** 2020-10-29

**Authors:** Chengyu You, Yuelin Du, Hui Wang, Lei Peng, Tangqiang Wei, Xiaojun Zhang, Xianhui Li, Anguo Wang

**Affiliations:** Department of Urology, Nanchong Central Hospital, The Second Clinical College, North Sichuan Medical College (University), Nanchong, China

**Keywords:** kidney neoplasm, laparoscopy, nephrectomy, surgical procedures, treatment outcomes

## Abstract

**Purpose:**

To summarize and analyze the current evidence about surgical, oncological, and functional outcomes between laparoscopic partial nephrectomy (LPN) and open partial nephrectomy (OPN).

**Materials and Methods:**

Through a systematical search of multiple scientific databases in March 2020, we performed a systematic review and cumulative meta-analysis. Meanwhile, we assessed the quality of the relevant evidence according to the framework in the Cochrane Handbook for Systematic Reviews of Interventions.

**Results:**

A total of 26 studies with 8095 patients were included. There was no statistical difference between the LPN and OPN in the terms of operation time (p=0.13), intraoperative complications (p=0.94), recurrence (p=0.56), cancer-specific survival (p=0.72), disease-free survival (p=0.72), and variations of estimated glomerular filtration rate (p=0.31). The LPN group had significantly less estimated blood loss (P<0.00001), lower blood transfusion (p=0.04), shorter length of hospital stay (p<0.00001), lower total (p=0.03) and postoperative complications (p=0.02), higher positive surgical margin (p=0.005), higher overall survival (p<0.00001), and less increased serum creatinine (p=0.002). The subgroup analysis showed that no clinically meaningful differences were found for T1a tumors in terms of operation time (p=0.11) and positive surgical margin (p=0.23). In addition, the subgroup analysis also suggested that less estimated blood loss (p<0.0001) and shorter length of hospital stay (p<0.00001) were associated with the LPN group for T1a tumors.

**Conclusions:**

This meta-analysis revealed that the LPN is a feasible and safe alternative to the OPN with comparable surgical, oncologic, and functional outcomes. However, the results should be applied prudently in the clinic because of the low quality of evidence. Further quality studies are needed to evaluate the effectiveness LPN and its postoperative quality of life compared with OPN.

## Introduction

For T1 (≤7 cm) renal masses, partial nephrectomy (PN) is the preferred surgical treatment, which is suggested by guidelines (1–3). On the one hand, PN is similar to radical nephrectomy in oncological safety (4, 5). On the other hand, PN protects kidney function better and reduces the incidence of cardiovascular diseases (4, 6). Although laparoscopic PN (LPN) is an enormous technical challenge and has a steep learning curve, it is obviously becoming a feasible alternative to open PN (OPN) with less blood loss, fewer complications, and comparable oncologic and functional outcomes (7–11).

With the development of laparoscopic techniques, the robotic technique has been frequently reported (12, 13). However, the robotic technology has not been fully popularized because of the limitations of economics or cognitions. Recently, hybrid transvaginal note nephrectomy also brought about widespread attention due to the superiority of sexual function, especially in the female population, but it needs further verification (14, 15). Therefore, the LPN is the first choice for primarily experienced centers because of better cost-efficacy (16).

There is always a lack of systemic evidence for LPN versus OPN even though the numbers of studies on it have increased recently. It is high time to perform a meta-analysis of outcomes for LPN versus OPN even though there are no randomized studies. Consequently, we conducted a systemic review and meta-analysis for LPN versus OPN, including surgical, oncological, and functional outcomes.

## Methods

The protocol of this review was registered prospectively (CRD42020178120) in the PROSPERO database (University of York, York, United Kingdom). The study was performed according to the preferred reporting items for systematic reviews and meta-analysis (PRISMA) statement (17).

### Literature Search and Study Selection

In April 2020, a comprehensively systematic literature search was conducted by using PubMed, the Cochrane Library, and Embase databases. The different search strategies were used for corresponding research engines, respectively. Search terms combined participant terms (kidney or renal neoplasm, kidney or renal cancer, kidney or renal carcinoma, kidney or renal tumor), intervention terms (partial nephrectomy or nephron-sparing surgery), and comparison terms (laparoscopic or laparoscopy, open). What is more, additional records were identified through manually searching references in the selected manuscripts or in the review articles. Literature searching imposed restrictions including being published in the English language and published from 2000 to 2020.

The studies focused on patients with kidney cancer and comparing surgical, oncological and functional outcomes between LPN and OPN were included. The studies involving patients with kidney tumor >7 cm were excluded to minimize the differences caused by the size of tumor. To eliminate discrepancies from the surgical approach, only the patients who underwent LPN were included. The studies that reported hand-assisted or robot-assisted laparoscopic technology were excluded. Meanwhile, letters, cases, reviews, conference abstracts, and studies that are irrelevant to the theme or lack complete data were excluded in order to enhance the feasibility and quality of the conclusions.

All included studies were assessed according to the methodological index for nonrandomized studies (MINORS) with a total of 24 points, which involves 12 items (18). In addition, the level of evidence of each study was assessed by the Oxford Centre of Evidence Based Medicine criteria (19). In addition, the risk of bias of each study included was independently assessed using the Risk of Bias in Non-Randomized Studies of Interventions tool (ROBINS-I) for comparative studies (20).

In addition, a subgroup analysis was performed in the patients with clinical T1a stage tumor to compare the two surgical techniques simply because the size of tumor is associated with surgical outcomes.

### Data Extraction

All outcomes of interest were collected in a piloted form, including the characteristics of selected studies, surgical, oncological, and functional outcomes. For the characteristics, the following items were included: author’s name, study design, number of patients, mean age, gender ratio, tumor location, tumor pathology, tumor size, and follow-up duration. The surgical outcomes included operation time, estimated blood loss (EBL), blood transfusion, length of hospital stay (LOS), and complications (total, intraoperative, and postoperative). The oncological outcomes contained positive surgical margin (PSM); recurrence; and survival results, including overall survival (OS), cancer-specific survival (CSS), and disease-free survival (DFS). The items of variations of estimated glomerular filtration rate (eGFR) and serum creatinine (sCr) were recorded for the functional outcomes. For survival data, we excavated data from Kaplan-Meier curve using Engauge Digitizer version 4.1 (http://digitizer.sourceforge.net/) for the studies without direct survival data.

The above two steps (literature search and data extraction) were completed by three of us (CY, YD, HW) independently. All disagreements were resolved by a senior author (AW) after public discussion.

### Data Analysis

The Review Manager software (RevMan) version 5.3 (the Cochrane Collaboration) was used for statistical analysis in our study. The mean difference (MD) and odds ratio (OR) were calculated for continuous and dichotomous variables, respectively, with 95% confidence intervals (CIs). In addition, the hazard radio (HR) with 95% CIs was used for CSS and DFS. We used special statistical methods for studies that presented merely continuous data as median and range values (21). The heterogeneity between studies was assessed by using the chi-squared and I- squared test. Random-effects models were used for cumulative analyses, which had high heterogeneity (I²>90%). Otherwise, fixed-effects models were used for analyses. Finally, *P* values of <0.05 were considered as a statistical significance for the meta-analysis.

What is more, the level of evidence for the outcomes was assessed using the framework in the Cochrane Handbook for Systematic Reviews of Interventions (22). A funnel plot was used to assess the risk of publication bias for outcomes that included at least 10 statistically significant studies.

## Results

**Figure 1:**
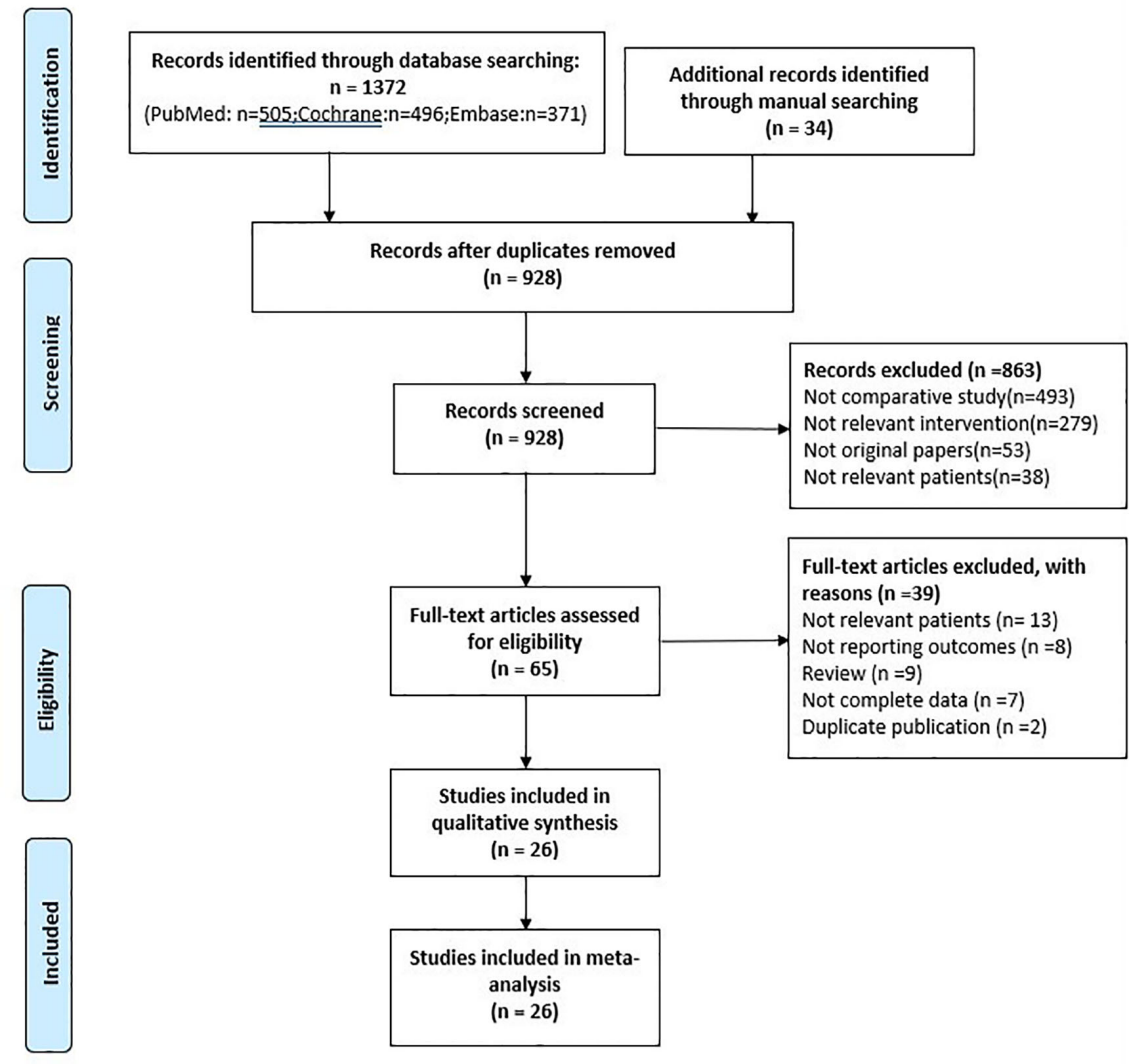
PRISMA flow diagram.

Initially, a total of 1406 studies were identified by our search strategy. First, 478 records were excluded because of duplication. Second, 863 studies were excluded that were irrelevant to our inclusion criteria by screening records. Third, 39 records of the remaining 65 were excluded by reading the full text (13 included irrelevant patients, 8 without reporting outcomes, 9 reviewers, 7 without complete data, and 2 duplicate publication). Finally, the remaining 26 studies were included with 8095 patients in our meta-analysis (**Figure 1**).

The characteristics of the included studies are shown in **Supplementary Table 1**. All the included studies—6 prospective studies (23–28) and 20 retrospective studies (22, 29–47)—were cohort observational studies with no randomization. There were 3292 and 4803 patients in the LPN and OPN groups, respectively. The mean ages ranged from 49.3 to 63.7 years and from 46.2 to 65 years in LPN and OPN, respectively. The mean MINORS scores of all the included studies were 11.9 (from 6 to 18). Twelve included studies (22, 27, 29, 31, 33, 36–38, 40, 42, 43, 46) were found to have a high risk of bias because of the selection of patients, performance bias, and observer bias of outcomes according to the ROBINS-I tool. The others had a moderate risk of bias (in **Supplementary Table 2**).

### Surgical Outcomes

**Figure 2:**
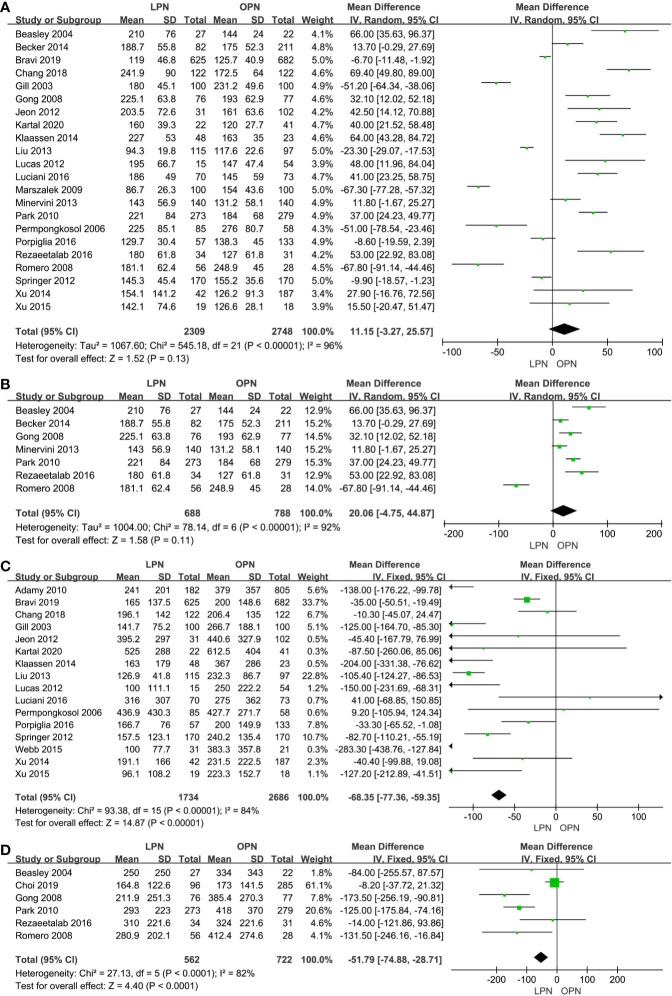
Forest plot and meta-analysis of operation time **(A)** and EBL **(C)**, the subgroup analysis of operation time **(B)** and EBL **(D)**.

There was no statistical difference between LPN and OPN for operation time (p=0.13, MD: 11.15 min, 95% CI: -3.27, 25.57, **Figure 2A**). Meanwhile, no clinically meaningful differences were found when T1a (p=0.11, MD=20.06 min, 95% CI: -4.75, 44.87, **Figure 2B**) was analyzed in subgroup analyses. The quality of evidence was low because of high heterogeneity and the potential of performance biases.

**Figure 3:**
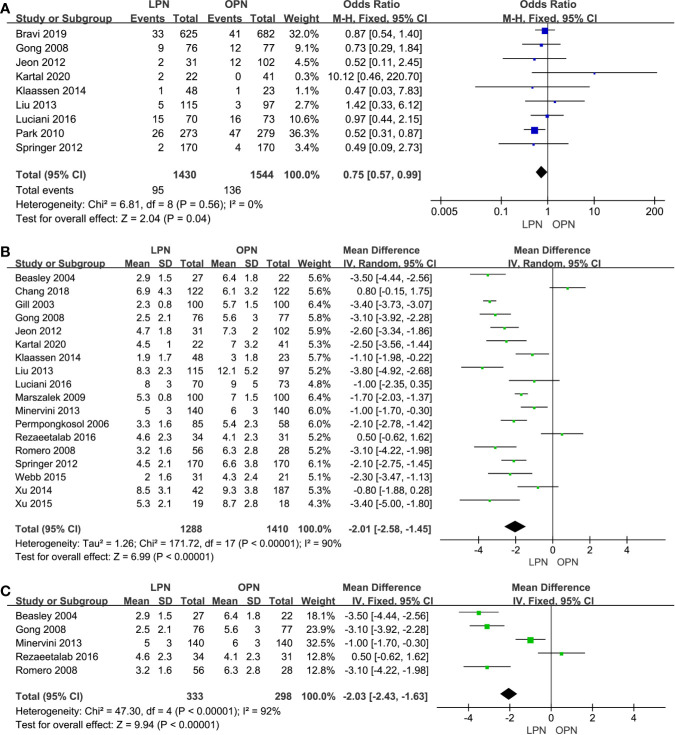
Forest plot and meta-analysis of blood transfusion rate **(A)** and LOS **(B)**, the subgroup analysis of LOS **(C)**.

Less EBL was associated with the LPN group in the total analysis (P<0.00001, MD: -66.16 mL, 95% CI: -74.56, -57.77, **Figure 2C**) and subgroup analysis (p<0.0001, MD: -51.79 mL, 95% CI: -74.88, -28.71, **Figure 2D**), respectively. Similarly, a lower blood transfusion rate was found in the LPN group (p=0.04, OR: 0.75, 95% CI: 0.57-0.99, **Figure 3A**). The quality of evidence for EBL and transfusion both were moderate.

For LOS, there was a statistically significant difference between the two surgical techniques (p<0.00001, MD: -2.01 days, 95% CI: -2.58, -1.45, **Figure 3B**). Moreover, subgroup analysis showed that a shorter LOS was related to LPN (p<0.00001, MD: -2.03 days, 95% CI: -2.43, -1.63, **Figure 3C**) for the clinical stage of T1a. The quality of evidence was judged to be moderate according to the Cochrane Handbook.

**Figure 4:**
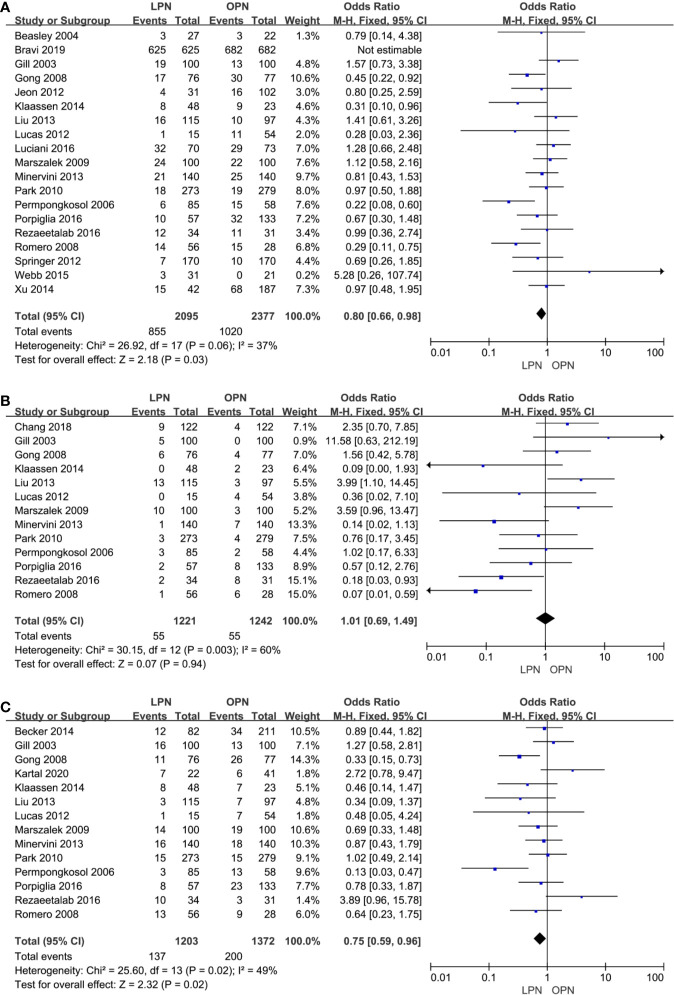
Forest plot and meta-analysis of total complications **(A)**, intraoperative complications **(B)** and postoperative complications **(C)**.

No clinically meaningful differences were found between two groups for term of intraoperative complications (p=0.94, OR: 1.01, 95% CI: 0.69, 1.49, **Figure 4B**). However, fewer complications were found in terms of both total (p=0.03, OR: 0.80, 95% CI: 0.66, 0.98, **Figure 4A**) and postoperative complications (p=0.02, OR: 0.75, 95% CI: 0.59, 0.96, **Figure 4C**). The quality of evidence was graded as low because of no classification of complications and the potential of performance, detection, and attrition biases influencing the estimate of effect.

### Oncological Outcomes

**Figure 5:**
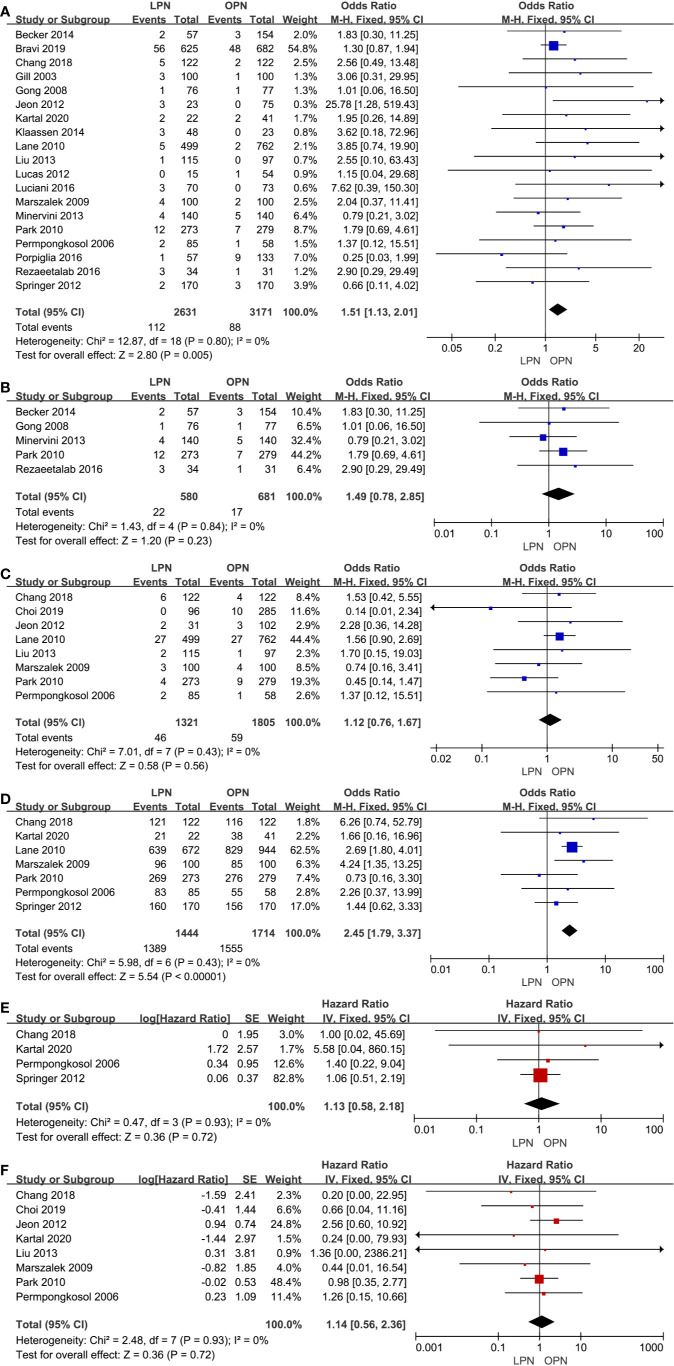
Forest plot and meta-analysis of oncological outcomes: PSM **(A)**, recurrence **(C)**, OS **(D)**, CSS **(E)**, and DFS **(F)**. The subgroup analysis of PSM **(B)**.

The higher PSM was in connection with the LPN (p=0.005, OR: 1.51, 95% CI: 1.13, 2.01, **Figure 5A**). Nonetheless, no statistically significant difference was found in subgroup analysis (p=0.23, OR: 1.49, 95% CI: 0.78, 2.85, **Figure 5B**). The quality of evidence was judged to be low because of selection bias and inconsistency of results from populations.

There was no clinically meaningful differences for recurrence between the LPN and OPN (p=0.56, OR: 1.12, 95% CI: 0.76, 1.67, **Figure 5C**). The quality of evidence was moderate.

For survival outcomes, there was a statistically significant difference between the two surgical techniques regarding OS (p<0.00001, OR: 2.45, 95% CI: 1.79, 3.37, **Figure 5D**). The quality of evidence was graded as very low because of the potential of selection bias and circumstantial evidence. Yet, no significant difference was found for terms of CSS (p=0.72, HR: 1.13, 95% CI: 0.58, 2.18, **Figure 5E**) and DFS (p=0.72, HR: 1.14, 95% CI: 0.56, 2.36, **Figure 5F**). The quality of evidence was low because of the small sample size, the potential of performance, and detection biases, respectively.

### Functional Outcomes

**Figure 6:**
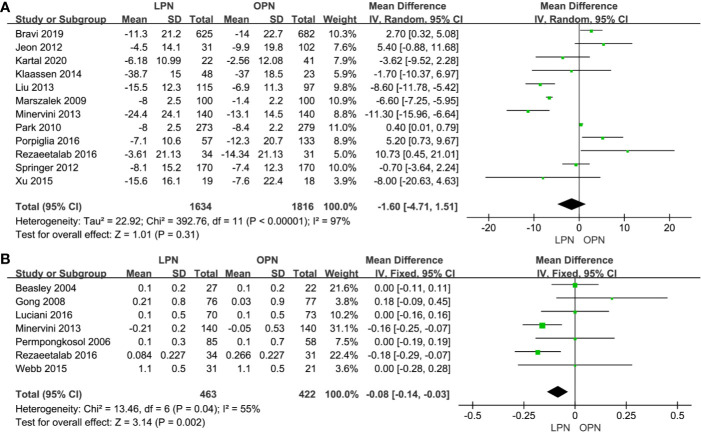
Forest plot and meta-analysis of functional outcomes: eGFR **(A)** and sCr **(B)**.

No statistically significant difference between the two surgical techniques regarding eGFR (p=0.31, MD: -1.60 mL/min/1.73m^2^, 95% CI: -4.71, 1.51, **Figure 6A**). There was a clinically meaningful difference between the two groups for term of sCr (p=0.002, MD: -0.08 mg/dL, 95% CI: -0.14, -0.03, **Figure 6B**). The quality of evidence was low because of high heterogeneity and the potential of performance bias, respectively.

### Publication Bias

**Figure 7:**
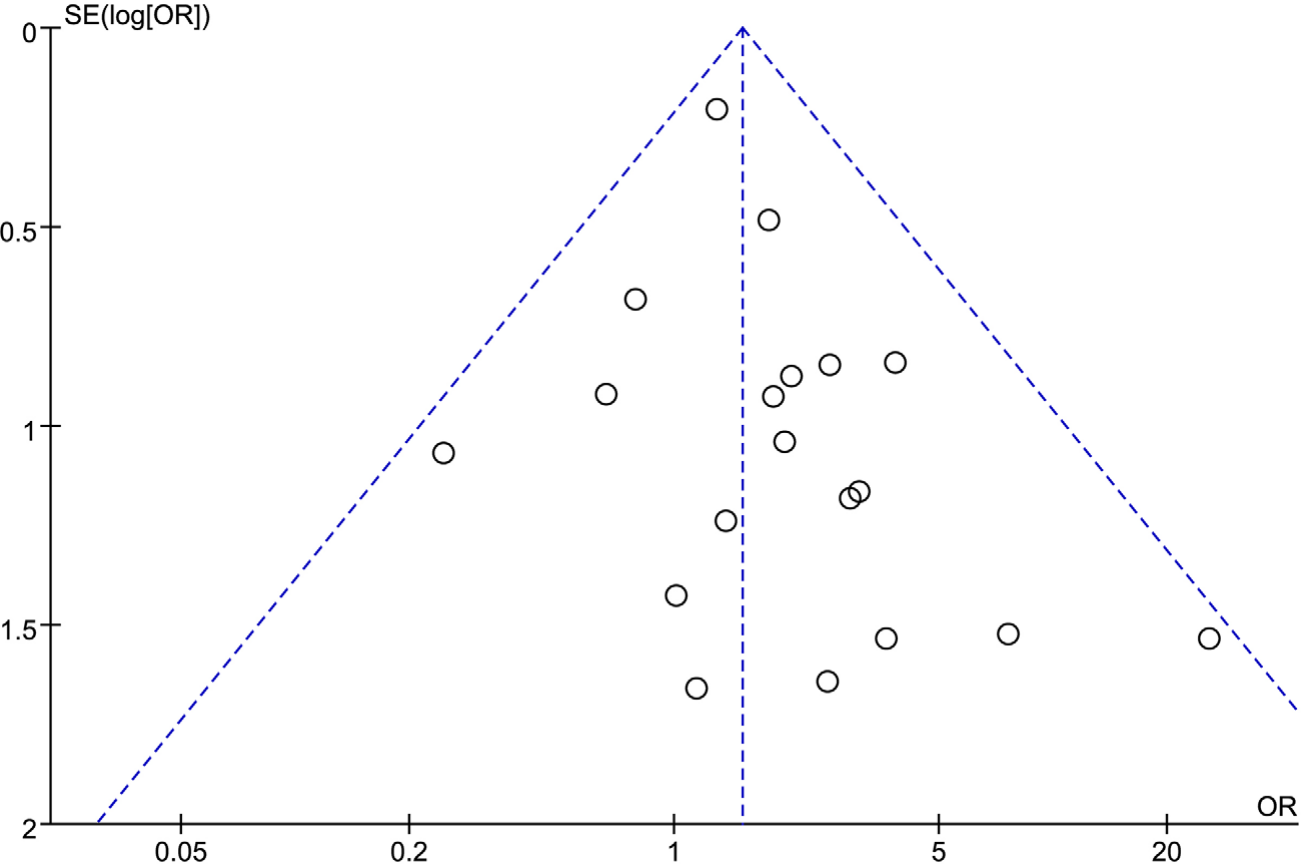
Funnel plot of PSM.

We analyzed possible publication bias generating funnel plots used for the evaluated comparisons of outcomes. There was apparent publication bias in most of the outcomes. For example, we present the funnel plot of PSM showing the obvious asymmetry (**Figure 7**).

## Discussion

We believed that there is a different effect on surgical, oncological, and functional outcomes between LPN and OPN. Due to lack of systematic evidence, we performed a meta-analysis and found several significant results after a systematic review of the literatures.

First, the results of surgical outcomes show that no significant difference was found pertaining to the operative time with high heterogeneity. Even so, some experienced centers report that shorter operative time has something to do with the LPN, which proves that the LPN has a great potential to transcend the OPN in terms of operative time for experienced centers (25, 39, 42, 43). Fu et al. prove that the retroperitoneal LPN has significantly less operating time the transperitoneal LPN (48). No subgroup analysis was performed because lack of sufficient data. Therefore, the tumor size, initial experience, and peritoneal access play main roles in the high heterogeneity. In addition, a lower volume (66.16 ml) of EBL is associated with LPN, which is not necessarily of clinical significance unless the total blood loss is sufficient to transfuse. Hence, we further analyzed the difference in intraoperative blood transfusion rate between the two surgical techniques and found that 15% risk of transfusion was reduced by LPN. It is pneumoperitoneum and superior vision that contribute to reduce blood loss providing precise dissection (23, 25, 34). A lower transfusion rate is associated with postoperative survival in patients with renal cell carcinoma after nephrectomy and reduces potential risk factors, such as hemolysis in the clinic (49, 50). What is more, results of LOS are 2.01 days shorter for LPN than OPN with high heterogeneity because of lower complications, smaller wounds, and more rapid recovery (23, 34, 44). We believe that the discharge criteria, postoperative care management, and characteristics of patients are associated with high heterogeneity. Due to the lack of scientific and strict classification of complications in most included studies and the lack of sufficient data, we only analyzed the complications classified into total, intraoperative, and postoperative complications according to the time of occurrence. Our cumulative analysis showed lower total and postoperative complications were related to the LPN compared with the OPN and showed no clinically meaningful differences were found for term of intraoperative complications. Marszalek et al. and Rezaeetalab et al. believe that shorter anesthesia and ischemia times are attributed to the lower perioperative complications, respectively (25, 28). On the other hand, some series thought the small difference in mean tumor size and tumor location were associated with fewer complications (26, 34, 35, 41). The fewer complications are beneficial to improve postoperative recovery and quality of life, which is more popular in the clinic.

Second, the analysis of oncological outcomes indicated that a higher PSM was found in the group of LPN. Subgroup analysis showed no significantly meaningful differences in term of PSM between two groups for T1a stage tumor. We believe that tumor size and learning curve play an important role in the discrepancy because the limited operation range of the laparoscope and less complete excision than open surgery was associated with high PSM for larger tumors. Current research suggests that a higher PSM is closely related to a higher incidence of local relapses, especially in large RCC, poorly differentiated, and/or more centrally located (51–53). However, the LPN and OPN accessed yield comparable in terms of recurrence, CSS, and DFS. It may be related to differences in pathological stage and follow-up time. In addition, we found that a high OS was associated with the LPN. Lane et al. thought that it had something to do with the renal functional outcomes (38). However, there is no clinical significance because of too many influences, such as underlying diseases and accidents.

Third, our results notably reveal that significant differences were found for postoperative change in sCr but not for postoperative changes in eGFR with moderate and high heterogeneity, respectively. The differences of patients’ characteristics, ischemia technique, and time are associated with heterogeneity. Marszalek et al. believe that the functional outcomes were closely related to intraoperative renal perfusion, caused by either arterial clamping or capnoperitoneum or capnoretroperitoneum (25). In addition, Bravi et al. suggest that surgical manipulation and suture/hemostatic techniques may affect early postoperative renal function (23). In addition, ischemia technique and time are related to postoperative renal function (26, 28). Subgroup analysis was not possible due to the lack of data. Recently, a systematic review proved that functional outcomes had something to do with ischemia technique, but none of the available ischemia techniques could be recommended over the other (54). Yet, a 0.08 mg/dL less increased sCr has no significant difference in the clinic.

Few studies focused on quality of life after PN. Becker et al. report that LPN and OPN were equivalent with postoperative quality of life, which needs further argument (31). For cost, current studies suggested that LPN is more cost-effective than OPN because of shorter LOS (55).

Although we performed this meta-analysis with the rigorous methodology of review and quantitative synthesis, some inherent limitations cannot be avoided. First, there were no prospective randomized controlled studies, which reduced the quality of evidence. Second, results should be applied carefully in clinical practice because of great heterogeneity in terms of operative time, LOS, and variations of eGFR. Third, some data were unsuitable to evaluate oncologic outcomes, including recurrence, OS, CSS, and DFS, because of insufficient follow-up period. Finally, there was evidence of the apparent publication bias. Computer-based literature searching could not include all relevant studies. Gray literature also could not be included.

## Conclusions

This meta-analysis revealed that the LPN is a feasible and safe alternative to the OPN with comparable surgical, oncologic, and functional outcomes. However, the results should be applied prudently in the clinic because of the low quality of evidence. Further quality studies are needed to evaluate effectiveness of LPN and its postoperative quality of life compared with OPN.

## Data Availability Statement

All datasets presented in this study are included in the article/**Supplementary Material**.

## Author Contributions

Conceived and designed the experiments: AW. Analyzed the data: CY, YD, LP, and HW. Contributed reagents/materials/analysis: YD, LP, TW, and XZ. Wrote the manuscript: CY and YD. All authors contributed to the article and approved the submitted version.

## Conflict of Interest

The authors declare that the research was conducted in the absence of any commercial or financial relationships that could be construed as a potential conflict of interest.

## Supplementary Material

Supplementary Table 1 Characteristics and quality assessment of included studies.
