# Supplementary material for: Laparoscopic Versus Open Partial Nephrectomy: A Systemic Review and Meta-Analysis of Surgical, Oncological, and Functional Outcomes
Source: Front Oncol. 2020 Oct 29;10:583979. doi: 10.3389/fonc.2020.583979 (PMC7658533; doi:10.3389/fonc.2020.583979)
Supplement: Supplementary Table 1 — Characteristics and quality assessment of included studies. [file Table_1.docx]

| Study | Study design | NO.of patients(LPN/OPN) | Mean Age(years) | | Gender(Male:Female) | | Tumor location(Right:Left) | | Pathology(benign: malignant) | | Tumor size^*^(cm) | | Follow-up duration^*^(mons) | | Level of evidence^**^ |
| --- | --- | --- | --- | --- | --- | --- | --- | --- | --- | --- | --- | --- | --- | --- | --- |
|  |  |  | LPN | OPN | LPN | OPN | LPN | OPN | LPN | OPN | LPN | OPN | LPN | OPN |  |
| Adamy et al | R | 182/805 | 60 | 60 | 114:68 | 480:325 | NA | NA | NA | NA | 3.1±1.9 | 3.1±1.7 | NA | NA | 4 |
| Beasley et al | R | 27/22 | 53.5 | 51.1 | 16:11 | 14:8 | 13:14 | 12:10 | NA | NA | 2.4±1 | 2.9±0.9 | NA | NA | 2b |
| Becker et al | R | 82/211 | 63.7 | 62 | 57:25 | 141:70 | NA | NA | 25:57 | 57:154 | 2(2-4) | 3(2-4) | NA | NA | 4 |
| Bravi et al | P | 625/682 | 63 | 65 | 421:204 | 438:244 | 315:310 | 374:308 | 99:478 | 98:540 | NA | NA | NA | NA | 2b |
| Chang et al | R | 122/122 | 53.5 | 53.8 | 69:53 | 66:56 | NA | NA | 14:108 | 13:109 | 2.7(1.9-4.3) | 2.5(2-4.5) | 60(46-70) | 64(52-77) | 2b |
| Choi et al | R | 96/285 | 53.1 | 54.6 | 74:22 | 212:73 | NA | NA | NA | NA | 2±0.9 | 2.8±1.4 | 52.8 | 52.8 | 4 |
| Gill et al | R | 100/100 | 65.1 | 58.8 | 58:42 | 67:33 | 58:42 | 46:54 | 30:70 | 85:15 | 2.8(2-4) | 3.3(3-4) | NA | NA | 2b |
| Gong et al | R | 76/77 | 60.1 | 59.7 | 35:41 | 42:35 | 37:39 | 43:34 | 22:54 | 17:60 | 2.87±0.81 | 2.45±0.87 | 21.7 | 20.6 | 2b |
| Jeon et al | R | 31/102 | 55.8 | 51.9 | 17:14 | 73:29 | 15:15 | 62:38 | 8:23 | 27:75 | 2.3±1.9 | 2.6±2 | 35±13.7 | 54.4±32.7 | 4 |
| Kartal et al | R | 22/41 | 52.7 | 58.2 | 14:8 | 25:16 | 13:9 | 21:20 | 3:19 | 3:38 | 4.75(4.2-7.5) | 5.1(4.1-7.4) | 62(27-78) | 54(37-78) | 4 |
| Klaassen et al | R | 48/23 | 56 | 62 | 30:18 | 15:8 | 24:24 | 13:10 | 12:36 | 2:21 | 3±1.9 | 3.8±1.1 | NA | NA | 4 |
| Lane et al | R | 672/944 | 60.3 | 61 | 395:277 | 626:318 | NA | NA | 173:499 | 182:762 | 2.5(1.7-3.2) | 3(2.2-4) | 50.4±26.4 | 67.2±30 | 4 |
| Liu et al | R | 115/97 | 51.2 | 53.1 | 72:43 | 59:38 | 60:55 | 48:49 | 20:95 | 24:73 | 3.23±1.42 | 3.09±1.32 | 29.3±14.4 | 31.2±12.6 | 4 |
| Lucas et al | R | 15/54 | 49.4 | 57.6 | 5:10 | 38:16 | NA | NA | 4:11 | 10:44 | 2.2±1.4 | 2.3±0.8 | 29(41.2) | 16.8(32.8) | 4 |
| Luciani et al | P | 70/73 | 62 | 63 | 42:28 | 51:22 | 35:35 | 39:34 | 12:58 | 23:50 | 3.5±1.4 | 3.6±2.3 | NA | NA | 2b |
| Marszalek et al | P | 100/100 | 62.3 | 62.5 | 60:40 | 60:40 | 54:46 | 53:47 | 19:81 | 34:66 | 2.8(2-3.2) | 2.9(2.3-3.5) | 44.4±2.4 | 42±2.4 | 2b |
| Minervini et al | P | 140/140 | 62.2 | 63 | 92:48 | 87:53 | 71:69 | 72:68 | NA | NA | 2.46±0.8 | 2.48±0.8 | NA | NA | 2b |
| Park et al | R | 273/279 | 54.6 | 53.1 | 191:82 | 207:72 | 107:87 | 135:130 | NA | NA | 2.1±0.8 | 2.3±0.9 | 17.8±13 | 28±20.9 | 2b |
| Permpongkosol et al | R | 85/58 | 58.2 | 57 | 61:24 | 39:19 | 40:45 | 31:27 | NA | NA | 2.4±1.1 | 2.9±0.9 | 40.4±18 | 49.68±28.84 | 4 |
| Porpiglia et al | P | 57/133 | 60 | 62.3 | 42:15 | 87:46 | 37:20 | 68:65 | NA | NA | 5(4.3-5.5) | 5(4.5-5.6) | NA | NA | 4 |
| Rezaeetalab et al | P | 34/31 | 50.3 | 54.8 | 23:11 | 23:8 | NA | NA | NA | NA | 3.4±1.3 | 3.7±1.3 | NA | NA | 2b |
| Romero et al | R | 58/28 | 58 | 57.4 | NA | NA | 28:28 | 12:16 | 11:45 | 4:24 | 3.1±0.9 | 3.2±1 | NA | NA | 4 |
| Springer et al | R | 170/170 | 55.6 | 56.1 | 112:58 | 117:53 | 78:92 | 72:98 | NA | NA | 2.8±1.9 | 2.9±1.4 | NA | NA | 2b |
| Webb et al | R | 31/21 | 55.53 | 53.6 | 19:12 | 14:7 | 13:18 | 5:16 | 5:26 | 2:19 | 2.7±0.92 | 4.22±1.34 | NA | NA | 4 |
| Hua Xu et al | R | 42/187 | 53.2 | 51.5 | 19:23 | 119:68 | 21:21 | 89:98 | 1:25 | 2:130 | 3.3±2.2 | 3.6±1.4 | NA | NA | 4 |
| Ben Xu et al | R | 19/18 | 49.3 | 46.2 | 14:5 | 13:5 | NA | NA | NA | NA | 3±1.1 | 3.5±1.5 | 37.8 | 37.8 | 4 |

P: prospective cohort; R: retrospective; LPN: laparoscopic partial nephrectomy; OPN: open partial nephrectomy; NA: not available;

^*^ The mean or median were used to show central tendency for tumor size and follow-up duration,and standard deviation or interquartile range were used for variation.

^**^ The Oxford Central for Evidence-based Medicine was used to assess.
