# Supplementary material for: Laparoscopic Versus Open Partial Nephrectomy: A Systemic Review and Meta-Analysis of Surgical, Oncological, and Functional Outcomes
Source: Front Oncol. 2020 Oct 29;10:583979. doi: 10.3389/fonc.2020.583979 (PMC7658533; doi:10.3389/fonc.2020.583979)
Supplement: Supplementary file 2 [file Table_2.docx]

| Study | Pre-intervention | | At intervention | Post-intervention | | | | Overall |
| --- | --- | --- | --- | --- | --- | --- | --- | --- |
|  | Confounding | Selection | Classification | Deviations | Missing | Measurement | Report |  |
| Adamy et al | H | M | L | M | M | M | L | H |
| Beasley et al | M | M | L | M | M | M | M | M |
| Becker et al | H | M | M | M | M | L | L | H |
| Bravi et al | M | M | L | M | M | L | L | M |
| Chang et al | M | L | M | L | M | M | M | M |
| Choi et al | H | H | L | M | M | M | M | H |
| Gill et al | M | M | M | L | M | L | L | M |
| Gong et al | M | M | L | M | M | M | M | M |
| Jeon et al | H | H | M | M | M | M | M | H |
| Kartal et al | M | H | L | M | M | M | M | H |
| Klaassen et al | H | H | M | M | M | M | M | H |
| Lane et al | M | H | M | M | M | M | M | H |
| Liu et al | M | M | L | M | M | M | M | M |
| Lucas et al | H | M | L | M | M | M | M | H |
| Luciani et al | M | M | L | M | M | M | L | M |
| Marszalek et al | M | L | L | M | M | M | M | M |
| Minervini et al | M | M | L | M | M | M | M | M |
| Park et al | M | M | L | M | M | M | M | M |
| Permpongkosol et al | H | M | L | M | M | M | M | H |
| Porpiglia et al | H | H | M | M | M | M | M | H |
| Rezaeetalab et al | M | L | L | M | M | M | M | M |
| Romero et al | H | M | L | M | M | M | L | H |
| Springer et al | M | M | L | M | M | M | L | M |
| Webb et al | M | M | L | M | M | M | M | M |
| Hua Xu et al | M | H | L | M | M | M | M | H |
| Ben Xu et al | M | M | M | M | M | M | M | M |

H: high risk of bias; M: moderate risk of bias; L: low risk of bias
